# Comparative Genome Analysis of Two *Bacillus pumilus* Strains Producing High Level of Extracellular Hydrolases

**DOI:** 10.3390/genes13030409

**Published:** 2022-02-24

**Authors:** Daria S. Pudova, Anna A. Toymentseva, Natalia E. Gogoleva, Elena I. Shagimardanova, Ayslu M. Mardanova, Margarita R. Sharipova

**Affiliations:** Institute of Fundamental Medicine and Biology, Kazan (Volga Region) Federal University, Kazan 420008, Russia; tojmencevaaa@mail.ru (A.A.T.); negogoleva@kpfu.ru (N.E.G.); ryukula@gmail.com (E.I.S.); mardanovaayslu@mail.ru (A.M.M.); marsharipova@gmail.com (M.R.S.)

**Keywords:** *Bacillus pumilus*, whole-genome sequencing, pan-genome, prophage regions, degradome, proteases

## Abstract

Whole-genome sequencing of a soil isolate *Bacillus pumilus*, strain 7P, and its streptomycin-resistant derivative, *B. pumilus* 3-19, showed genome sizes of 3,609,117 bp and 3,609,444 bp, respectively. Annotation of the genome showed 3794 CDS (3204 with predicted function) and 3746 CDS (3173 with predicted function) in the genome of strains 7P and 3-19, respectively. In the genomes of both strains, the prophage regions Bp1 and Bp2 were identified. These include 52 ORF of prophage proteins in the Bp1 region and 38 prophages ORF in the Bp2 region. Interestingly, more than 50% of Bp1 prophage proteins are similar to the proteins of the *phi105* in *B. subtilis*. The DNA region of Bp2 has 15% similarity to the DNA of the *Brevibacillus Jimmer* phage. Degradome analysis of the genome of both strains revealed 148 proteases of various classes. These include 60 serine proteases, 48 metalloproteases, 26 cysteine proteases, 4 aspartate proteases, 2 asparagine proteases, 3 threonine proteases, and 2 unclassified proteases. Likewise, three inhibitors of proteolytic enzymes were found. Comparative analysis of variants in the genomes of strains 7P and 3-19 showed the presence of 81 nucleotide variants in the genome 3-19. Among them, the missense mutations in the *rpsL*, *comA*, *spo0F* genes and in the upstream region of the *srlR* gene were revealed. These nucleotide polymorphisms may have affected the streptomycin resistance and overproduction of extracellular hydrolases of the 3-19 strain. Finally, a plasmid DNA was found in strain 7P, which is lost in its derivative, strain 3-19. This plasmid contains five coding DNA sequencing (CDS), two regulatory proteins and three hypothetical proteins.

## 1. Introduction

The genus *Bacillus* is defined as Gram positive, aerobic or facultative anaerobic, motile (peritrichous flagella) and endospore-forming rod-shaped microorganisms [1]. Members of the *Bacillus* genus have been isolated from diverse habitats including soil, plant tissues [2], marine sediments [3] and extreme environmental conditions [4]. They could provoke food poisoning [5], animal and human diseases [1,6]. Many *Bacillus* species synthesize a wide variety of metabolites with antimicrobial activity (antibiotics, antimicrobial peptides), extracellular proteins (mainly proteases, lipase, amylase, cellulose, etc.) [7]. The U.S. Food and Drug Administration (FDA) gave the GRAS-status (the acronym for “generally recognized as safe”) for *B. subtilis*, *B. licheniformis* and *B. pumilus* species [GRAS Notice Inventory|FDA]. 

The genomics of *B. pumilus* species are not studied extensively compared to model organism *B. subtilis*. The NCBI database already includes 17 complete circular genomes and 156 genomes in scaffold/contigs level of *B. pumilus* strains (September 2021). The known strains of *B. pumilus* show high resistance to environmental parameters [2,4,8]. The *B. pumilus* SAFR-032 strain exhibits high survival rates under exposure to outer space conditions in experiments onboard the International Space Station (ISS) [4]. The rhizobacterium *B. pumilus* ZB201701 is able to stimulate plant growth and their resistance to drought and salinity [2]. Strain PDSLzg-1 isolated from oil-contaminated soil shows the ability to degrade hydrocarbons [9]. Isolated from the human gastrointestinal tract, the UAMX strain is able to metabolize various carbon sources, shows resistance to low pH values (pH = 3.0), and the presence of bile salts stimulates its growth [8].

In this study, we used the genomes of 7P and 3-19 strains to study genomic features unique to these strains and phenotypes. *B. pumilus* 7P is a wild-type strain isolated from the soil of the Republic of Tatarstan (Russia) as a producer of extracellular hydrolytic enzymes—RNase (Patent 587156) [10]. Based on the 7P strain, a probiotic has been developed as a feed additive for poultry [11]. To improve the RNase production of isolate 7P, the classical approach of conferring the resistance against antibiotics was applied. In this method, antibiotics able to repress the gene transcription, i.e., rifampicin, streptomycin, gentamicin or geneticin, or to disorder mRNA translation, such as erythromycin, are used to select spontaneous mutants which may increase the production of secondary metabolites and enzymes [12,13]. *B. pumilus* strain 3-19 is a 7P derivative obtained by inoculating the 7P strain on a nutrient medium supplemented with streptomycin (up to 500 μg/μL) and showed increased activity of extracellular hydrolase: RNase, phosphatase and proteases (Table S1) (Patent RU 2384619). On the basis of *B. pumilus* 3-19 RNase, a broad-spectrum antitumor drug is being developed [14]. Due to their ability to secrete various extracellular proteins, *B. pumilus* bacteria are a promising platform for the production of commercial enzymes. For instance, proteases from *B. pumilus* 3-19 (subtilisin-like serine protease, glutamyl endopeptidase and metzincine metalloprotease) have thrombolytic and anticoagulant activity, degrade β-amyloid peptide and disrupt biofilms of pathogenic microorganisms [15,16,17]. Unlike *E. coli* cell factories, Gram-positive bacteria do not synthesize endotoxins, have a well-developed secretion system, which greatly facilitates the production of protein preparations [18]. However, the low efficiency of transformation of *Bacilli* (except *B. subtilis* 168) is an obstacle to obtaining a higher yield of the target enzyme. In this study, the genomes of strains 7P and 3-19 were analyzed in order to clarify the genomic similarities and differences between these two strains and enable the effective utilization of these strains for the production of biotechnologically relevant enzymes.

## 2. Materials and Methods

### 2.1. B. pumilus Strains and Total DNA Extraction

Bacterial strains *B. pumilus* 7P (soil isolate, WT) and *B. pumilus* 3-19 (a strain with resistance to streptomycin) were used. The 7P strain was isolated from the soil of the Republic of Tatarstan (Russia) and identified on its ability to produce ribonuclease (binase). Both 7P and 3-19 strains were used for genomic DNA isolation and sequencing library preparation.

*B. pumilus* cultures were incubated at 37 °C for 14 h in an aerobic atmosphere. For bacterial cultivation, LB (Lysogeny broth and Lysogeny agar) medium (1% tryptone, 0.5% yeast extract, 0.5% NaCl, pH 8.5) was used. High molecular weight bacterial DNA was extracted using phenol/chloroform method [19]. The cultures of *B. pumilus* 7P and 3-19 strains were grown up to OD_600_ = 1.0. The cell pellet was resuspended in TEN buffer (10 mM Tris-HCl, pH 8.0; 10 mM EDTA; 150 mM NaCl) and for cleaving of bacterial cell wall lysozyme was added. RNase (20 mg/mL), SDS (10%), and proteinase K (20 mg/mL) were used for the enzymatic digestion of proteins and nonnucleic acid cellular components. DNA extraction was performed using phenol and a mixture of chloroform:isoamyl alcohol (24:1). The aqueous phase was transferred into ice-cold ethanol and stored at 4 °C. The quantity and quality of the purified DNA were measured by NanoPhotometer NP80 (Implen, Westlake Village, CA, USA) and by electrophoresis on a 1.5% agarose gel, respectively.

### 2.2. Genome Sequencing and Assembly

In order to obtain a complete genome, we sequenced high molecular weight DNA using Oxford Nanopore MinION (Great Britain). Reads obtained by Oxford Nanopore technology were combined with previously obtained reads by means of 454 GS Junior Roche pyrosequencing and the 200-bp chemistry Ion Torrent PGM platform [20,21]. Assessment of the quality of the data sequencing is performed using FastQC v. 0.11.3. Reads with a quality value of Q < 20 were excluded from further analysis by Trimmomatic v. 0.36 [22]. Each of the two genomes were assembled de novo by SPAdes v.3.12.0 [23]. The quality of assemblies were assessed using metrics implemented in QUAST [24]. Whole genomes have been deposited in the DDBJ/EMBL/GenBank under accession numbers CP058911.1 for the 7P strain and CP054310.1 for the 3-19 strain.

### 2.3. Genome Annotation and Comparative Analysis

The genomes were annotated using the NCBI Prokaryotic Genomes Annotation Pipeline and Prokka v. 1.11. For functional annotation IMG/M (The Integrated Microbial Genomes with Microbiome Samples) servers [25] were used. The available 17 complete genomes of *B. pumilus* were used for comparative analysis. Genomes and their accession numbers are presented in Table S2. A search for closely related strains was established using average nucleotide identity (ANI). ANI analysis was carried out by JspeciesWS [26], using the MUMmer algorithm. The heatmap was generated in R Environment v. 3.3.1, using “gplots” package. Multiple genome alignment was performed by BRIG [27] and MAUVE [28]. Orthology analysis for calculating the core genome was performed using Proteinortho [29]. Proteinortho was used for the identification of groups of orthologous proteins based on protein sequence similarities. The results of orthology analysis were visualized on a Venn diagram, where orthologous groups were treated as entities. Thus, an intersection area on a Venn diagram for given *B. pumilus* strains indicates a number of orthologous groups containing proteins from given strains genomes.

### 2.4. Plasmid and Phage Regions Prediction

An analysis of phage regions in the *B. pumilus* 7P/3-19 genomes was conducted using PHAge Search Tool—Enhanced Release (PHASTER) tool [30]. Visualization of prophage regions was carried out by Easyfig v 2.2.2 program [31]. The plasmid sequence in the *B. pumilus* 7P genome was predicted using BLASTn (NCBI) comparative analysis. The sequence of pDA7 plasmid has been deposited in GenBank under accession number CP076555.1. Extraction of plasmid was performed by GeneJET Plasmid Miniprep Kit (Thermo Fisher Scientific, Waltham, Massachusetts, USA). The presence of plasmid DNA was verified by electrophoresis on a 1.5% agarose gel.

### 2.5. Classification of Proteases Family

*B. pumilus* genomes FASTA (.faa) format files with all proteins, which were received by Prokka v1.11 program, were used for the study of proteases. Proteolytic enzymes were identified and classified using the MEROPS database (12.3; September 2020) [32]. The results were filtered by E-value: the protease was considered inactive if the E-value was greater than e^−10^. The subcellular localization of all annotated proteases was identified by SignalP v. 4.1 [33], TMHMM server v. 2.0 [34] and confirmed by further alignment in UniProt [35]. The protease sequences of the two strains were compared by Easyfig v 2.2.2 program, using the BLAST algorithm.

### 2.6. Variants Calling

Raw reads of strain 3-19 were mapped to the *B. pumilus* 7P genome using the Bowtie2 aligner [36]. The SAM alignment file was converted to BAM format using SAMTools v. 0.1.19 [37]. To invoke the variants were used SAMTools’ mpileup and BCFtools [37]. The effect of the changes was predicted using the SNP effect predictor (SnpEff) v. 4.0 [38]. The resulting variants were filtered by QUAL > 100.0, and mutations with a LOW contribution (synonymous amino acid substitutions) were removed from the study.

## 3. Results and Discussion

### 3.1. Genome Assembly of B. pumilus Strains 7P and 3-19

Prior to this study, comparative analysis of draft genomes of strain 7P and it’s streptomycin-resistant derivative 3-19 showed that *B. pumilus* 3-19 became streptomycin-resistant due to a mutation of the S12 protein of 30S ribosomal subunit, RpsL^K56N^ [39]. Here, further analysis of the genomes of *B. pumilus* strains 7P and 3-19 was carried out by complete sequencing, correct completion and circularization of genomes. Complete assemblies of the genomes of strains 7P and 3-19 were obtained with a total length of 3,609,117 bp and 3,609,444 bp, respectively (Table 1). Genomes were annotated using the NCBI Prokaryotic Genomes Annotation Pipeline. Both strains have genomes with an average GC content of ~42%, which corresponds to the GC content of the remaining *B. pumilus* genomes. In the genomes, 3564 (for 7P strain) and 3569 (for 3-19 strain) protein-coding sequences (CDSs) were identified. 

Using BLASTn (NCBI) comparative analysis, the plasmid sequence pDA7 was predicted in the 7P genome. The pDA7 plasmid has 6019 bp showing the highest similarity (99%) to pBP-33-3 plasmid from *B. pumilus* 33-3 with 6432 bp. The pBP-33-3 plasmid contains ten CDSs including RNA polymerase-associated proteins (RapA, RapAB), replication initiator protein (Rep), DNA-binding and hypothetical proteins. Annotation of pDA7 plasmid however showed the presence of five CDSs including response regulator aspartate phosphatase A (RapA), replication initiator protein (Rep) and three hypothetical proteins. Homological sequence of pDA7 was not found in the 3-19 genome. The presence of pDA7 in *B. pumilus* strain 7P was verified by plasmid extraction (Figure S1).

### 3.2. Phylogenetic Classification of B. pumilus Strains 7P and 3-19

Based on the morphological, biochemical and physiological characteristics, both the 7P and the 3-19 isolates were primarily identified as members of *B. intermedius* species. However, in the international databases (GenBank, EMBL) and in Bergey’s Manual of Systematic Bacteriology, there are no *B. intermedius* species. Thus, to understand the phylogenetic position of 7P/3-19 isolates, the 16S rRNA gene sequence analysis was performed. The 7P/3-19 isolates showed 99% homology with *B. pumilus* group [40]. The results of 16S rRNA gene sequencing were submitted to GenBank and are available under Accession No HQ650161.1 for 3-19 strain and JX129390.1 for 7P strain. To find the closest genomes to *B. pumilus* strains 7P and 3-19, we compared the genomes of these strains with other whole-genome sequenced *B. pumilus* strains. For comparisons and alignments, we included 17 available whole-genome sequences from *B. pumilus* (accessed September 2021, Figure 1). Average Nucleotide Identity (ANI) values between all organisms are represented in Table S3. The heatmap shows that *B. pumilus* strains formed several clusters (groups). Both strains 7P and 3-19 showed over 98% homology with strains ONU 554, ZB201701, PDSLzg-1 and EB130. The genome of *B. pumilus* ONU 554 (CP060799.1) strain showed the maximum ANI value—99.5%. Four strains, namely, TUAT1, MTCC B6033, SH-B11, and C4, formed a separate cluster that had less than 95% homology with other strains of *B. pumilus*. In a previous study, it has been reported that three strains, namely TUAT1, MTCC B6033 and SH-B11 cluster with other *B. altitudinis* [41]. Our findings confirm this conclusion and place the C4 strain under *B. altitudinis*.

**Figure 2:**
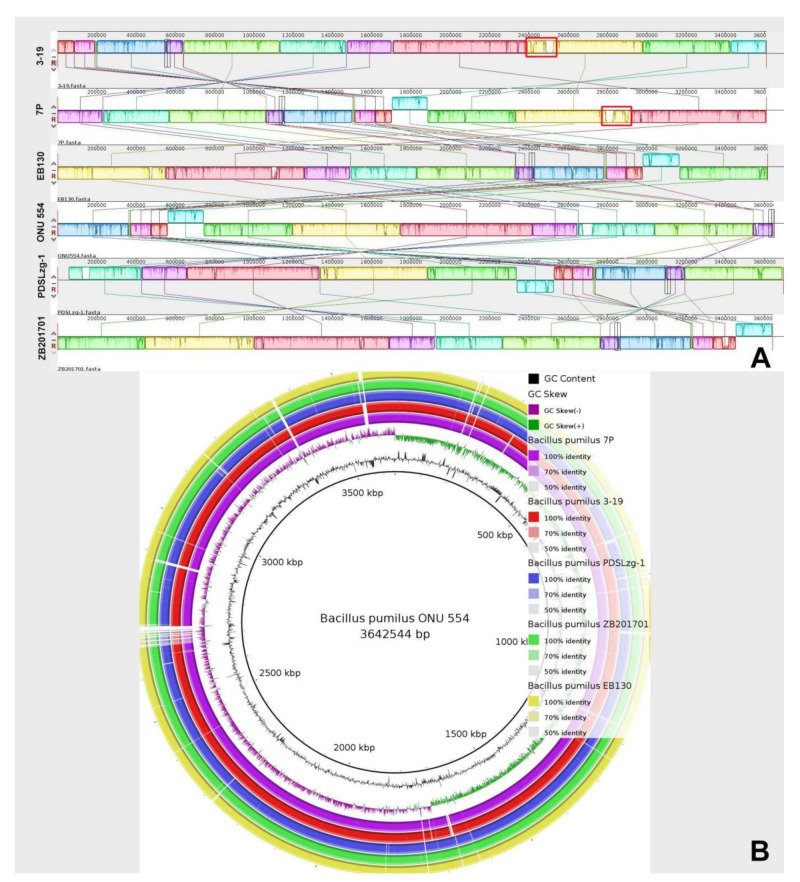
Multiple genome alignment of *B. pumilus* strains 7P and 3-19 with other whole-genome sequences *B. pumilus*. the nearest genomic neighbors. (**A**) Genome alignment by MAUVE. White areas specify the unique sequences of the genome. (**B**) Circular genomic maps of *B. pumilus* strains. Color saturation indicates the homology rate, blanks show the absence of similarity.

We performed multiple genome alignment of the *B. pumilus* strains in relation to strains closest in homology using MAUVE and BRIG. MAUVE analysis showed that genomes have a high content of homologous regions, which are located in the same sequence. The regions of the 7P and 3-19 strain genomes, which differ from the other closely related genomes, mainly contain proteins associated with prophages (Figure 2A). Circular comparison of *B. pumilus* genomes by BRIG also showed high structural homology of *B. pumilus* strains that belong to the same cluster (based on ANI analysis). Moreover, strains 7P and 3-19 contain similar regions that differ from other analyzed strains (Figure 2B). To better understand the similarities and differences of these regions, annotation and the function of these annotated regions must be considered.

### 3.3. Functional Prediction of the Annotated Genes

**Figure 3:**
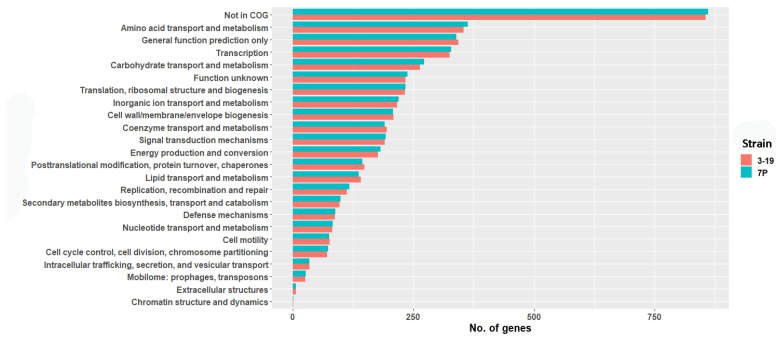
Comparative analysis of COG categories between 3-19 and 7P strains.

For genome annotation, the IMG server was used. In this way, 3794 protein-coding genes (3204 with function prediction) were annotated for the 7P strain. Likewise, 3746 protein-coding genes were annotated for strain 3-19 from which 3173 were annotated with their putative function. Thus, using the IMG server annotation, more protein-coding sequences were obtained, compared to PGAP in NCBI. The predicted protein sequences were compared to the COG database using BLASTp. A total of 78% of the proteins for both strains were classified in at least one COG category (Figure 3).

The main categories of proteins are encoded by housekeeping genes (Figure 3). Among proteins of the cell motility category flagellar hook-associated proteins, chemotaxis and competence proteins (ComGE, GD, GB, GA) were identified. In the 7P strain genome, 27 (26 for 3-19 strain) proteins associated with Mobilome phages: prophages, transposons, and 99 (97 for 3-19 strain) proteins—with Secondary metabolites biosynthesis, transport and catabolism were found. The Defense mechanisms category accounts for 2.4% of the total CDSs (Figure 3). Known strains of *B. pumilus* are highly resistant to UV radiation and hydrogen peroxide, which may explain the detection of viable *B. pumilus* spores in hostile environments such as the inner basalt surfaces of the Sonoran Desert and spaceships [4,42]. This stress tolerance can be a major benefit for improving commercial production strains of *B. pumilus*. In the genomes of 7P and 3-19 strains, we found genes of the Defense mechanisms category, whose products confer antibiotic resistance (aminoglycoside 3-N-acetyltransferase, bacitracin transport system permease protein, beta-lactamase class D, chloramphenicol O-acetyltransferase type A, CubicO group peptidase, β-lactamase class C family, glycopeptide antibiotics resistance protein), oxidative stress resistance (Ohr subfamily peroxiredoxin, glutathione peroxidase), acetoin utilization, tellurite and copper resistance, sporulation (stage III sporulation protein AF, AbrB family transcriptional regulator (stage V sporulation protein T)). The presence of ribonuclease toxin of YeeF-YezG toxin-antitoxin module and antitoxin component YwqK of YwqJK toxin-antitoxin module was detected. Polymorphic toxins belong to a family of toxins produced by bacteria that help restrict the growth of competitors, facilitate the selection of relatives, and form the bacterial community [43]. It has been shown that toxins in these systems (for example, YeeF) are nucleases that have RNase or DNase activity and are neutralized by the corresponding antitoxin [44]. Thus, based on the results of the genome annotation of 7P and 3-19 strains, we identified housekeeping genes, genes encoding prophage proteins (27 proteins for 7P and 26 proteins for 3-19), as well as genes for resistance to antibiotics, oxidative stress, rare metals, sporulation and toxins with nuclease activity.

### 3.4. Comparative Genomic Analysis of B. pumilus Strains

**Figure 4:**
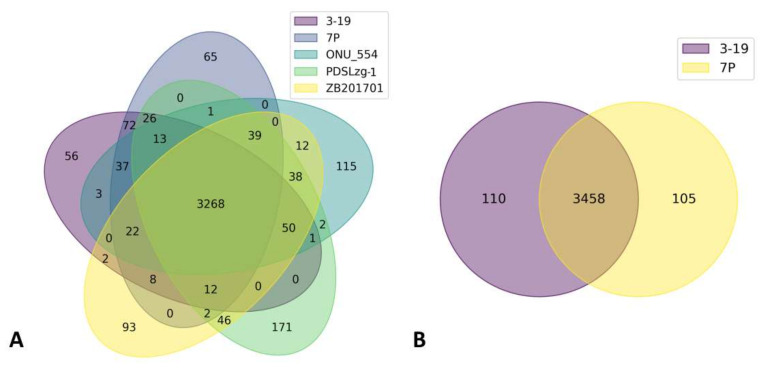
Comparative analysis of orthological set of proteins among five closely related *B. pumilus* strains (**A**) and two 7P and 3-19 strains (**B**).

To obtain an overall estimate of the set of genes for the species under study, comparative genomic analysis was carried out. The orthologous proteins of the five *B. pumilus* strains that have the closest phylogenetic similarity were compared (Figure 4A).

A total of 3268 proteins formed the core genome. Moreover, 72 orthologous proteins are unique for 7P and 3-19 and are absent in other closely related strains (Table S4). The presence of autolysins, integrases, and transposases genes was verified indicating the presence of prophage regions in the genomes of 7P and 3-19 strains. Among the common CDSs in 7P and 3-19, a set of regulatory proteins were present including RapH, a phosphatase inhibitor, and host-nuclease inhibitor Gam family protein, ImmA/IrrE family metallo-endopeptidase. The Gam protein is found in many bacterial species as part of a putative prophage. Biochemical studies have shown that the Gam protein exhibits DNA binding characteristics similar to those of the eukaryotic Ku protein and plays a key role in certain transposition events [45]. The anti-repressor ImmA is also found in many mobile genetic elements, in conjunction with the ImmR repressor. It was shown that the homolog of the anti-repressor ImmA, encoded by the *phi105 B. subtilis* phage, is required for inactivation of the *phi105* (homologue ImmR). ImmA-dependent proteolysis of ImmR repressors may be a conservative mechanism for the regulation of horizontal gene transfer [46]. As a result of comparing the genomes of two strains 7P and 3-19, 105 and 110 unique CDS were identified, respectively (Figure 4B). When comparing the functions of these proteins, it was found that 92 proteins have the same function, but differ in structure, which may be the result of inaccurate sequencing or genome assembly (Table S5). As a result, 18 and 13 unique CDSs were identified for strains 3-19 and 7P. Blast analysis of these proteins did not reveal the presence of sequences that could affect the overproduction of hydrolases in strain 3-19. Thus, it can be noted that there are no significant differences in the number of CDSs in the genomes of strains 7P and 3-19. Altogether, there are no large deletions or gene amplifications in the genome of 3-19 due to classical mutagenesis or activation of the prophages as well as mobile elements.

### 3.5. Genome-Based Identification of Prophage Regions

Analysis and prediction of dynamic parts of bacterial genomes (plasmids, integrative and conjugative elements (ICE), (pro)phages) is an important task of genomic annotation. It is known that many biotechnological bacterial cultures are infected by bacteriophages. That is why knowing the content of such elements will contribute to a better understanding of the diversity of unknown genes, probable resistance, pathogenicity and evolutionary process. Prophage regions identified in genomes can be classified as ‘intact’ or ‘incomplete’. Incomplete prophage regions are considered defective prophages. Defective prophages do not have complete structural prophage genes in comparison to the active, functional phages. However, defective prophages often carry genes that are beneficial to the host (genes of recombination, virulence, stress resistance, or toxins that can inhibit the growth of competing bacteria in the environment) [47].

**Figure 5:**
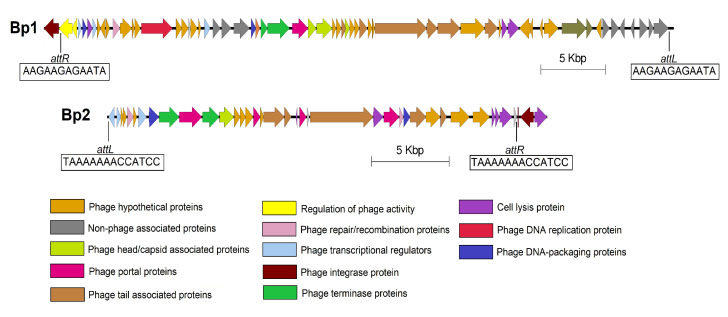
Phage regions of *B. pumilus* 3-19. The colors displayed basic phage and non-phage-associated proteins.

Two regions were annotated as prophage regions in genomes of 7P and 3-19 by PHASTER (Figure 5). The first prophage region (Bp1) had more than 90% score (intact prophage), second (Bp2)—had score less than 70% (incomplete prophage). PHASTER provided information about the length, GC content, and showed the presence and sequence of attachment sites. No significant differences were found between the prophage regions of strains 7P and 3-19. The Bp1 prophage length was ~48 Kbp, the Bp2 prophage length was ~30 Kbp. It should be noted that the GC content of predicted phage regions is different from the GC content of the whole bacillary genome (42%)—38% for Bp1 and 41% for Bp2. 

The first ‘intact’ prophage region (Bp1) has phage-like CDSs and non-associated proteins (iron chaperone, undecaprenyl-diphosphatase, GNAT family N-acetyltransferase, collagen-like protein, Fur-regulated basic protein FbpA). Among phage-like proteins genes of recombination/repair (host-nuclease inhibitor Gam family protein), transcription (ArpU family transcriptional regulator, helix-turn-helix family domain Xre family transcriptional regulator), termination (terminase large subunit, phage terminase small subunit P27 family), replication (DNA primase), DNA packaging (HNH endonuclease) were detected in the Bp1 phage region. The phage structural genes encode head and capsid structure proteins (phage head closure protein, phage major capsid protein, HK97 family phage prohead protease), tail/neck structure proteins (phage tail proteins, phage gp6-like head-tail connector protein, phage tail tape measure protein), portal proteins and proteins of lytic cycle (N-acetylmuramoyl-L-alanine amidase, holin). The Bp1 phage also has an integrase that enables the integration of phage genetic material into the DNA of the host. CI repressor is localized upstream from the integrase gene. This CI protein represses phage induction and retains phage in a lysogenic state. It is known that repressor proteins provide immunity to the infected strain against superinfection [48]. Next to the CI repressor is the ImmA/IrrE family metallo-endopeptidase gene, which is also responsible for the regulation of prophage activity [46]. More than 50% of the Bp1 proteins (31) are similar to proteins of bacillary *phi105* phage. This temperate phage was identified in the genome of *B. subtilis* 168. It is incapable of generalized transduction, but all three types (prophage, vegetative and mature DNA) of its DNA show specific characteristics in transfection. This moment makes the *phi105*—*B. subtilis* 168 system very useful for studies on the mechanism of transfection [49,50]. It is also known that the presence of this prophage is associated with a low transformation efficiency of *B. subtilis* bacteria [51] and repression of the mechanism of horizontal gene transfer [46]. Two proteins (antitoxin YezG family protein, ribonuclease YeeF family protein) of the toxin-antitoxin system (TAs) were defined by PHASTER as phage proteins in Bp1 prophage. TAs are “selfish” two-gene modules, which are contained in some mobile elements, embedded in the host genome. After cell division, the toxin component kills a cell, which does not receive the TAs-encoding proteins, so that only TAs-containing daughter bacteria survived [52]. This system probably could act similarly to phage holins, which may have been their primary function when the prophage was still functional [53].

The “incomplete” Bp2 phage region contains genes which responsible for the lytic cycle (N-acetylmuramoyl-L-alanine amidase, phage holin, endolysin, LysM peptidoglycan-binding domain-containing protein), termination (terminase, PBSX family phage terminase large subunit), integration, DNA packaging (putative single-stranded DNA binding protein), transcription (helix-turn-helix transcriptional regulator) and reparation/recombination (endodeoxyribonuclease RusA, recombination protein). Genes of structural proteins such as tail proteins (phage tail tube protein, phage tail sheath subtilisin-like domain-containing protein), portal proteins, baseplate associated protein, capsid protein were also found in the Bp2 region. Bp2 prophage showed a similarity (15%) with *Brevibacillus* phage Jimmer. Thus, the identification of prophage regions in the genomes of strains 7P and 3-19 confirms the results of the structural comparative analysis of genomes. The plausible consequence of the presence of prophage regions in studied strains is a negative effect on the formation of competence state and horizontal gene transfer. 

### 3.6. Genomic Analysis of B. pumilus Proteases and Protease Inhibitors

*Bacillus* species are famous expression hosts for secreting and producing foreign proteins. Their potential as biotechnological hosts is greatly determined by the amount of extracellular proteolytic enzymes which can degrade heterologous proteins. Construction of protease-free bacillary strains or screening of natural species with low extracellular enzymes production levels is an actual task of biotechnology [54]. Proteases are involved in critical processes such as cell behavior, proliferation, survival, production of signaling peptides, DNA packing, genetic competence, protein secretion, processing of proteins, preventing autolysis, supplying amino acids for growth via degradation of extracellular proteins etc. [55] Moreover, a growing interest in the identification and functional characterization of the complete set of proteases produced by cells (degradome) is clarified by their perspective biotechnological and clinical application. Here, we performed the first genomic analysis of the *B. pumilus* degradome.

Both *B. pumilus* genome sequences (7P and 3-19) were searched for the presence of proteases by using the MEROPS database (12.3; September 2020). This database is an information resource for peptidases and the proteins that inhibit them [32]. We analyzed the distribution of the 148 annotated proteases within different catalytic classes of proteolytic enzymes. Serine proteases are the most abundant proteolytic enzymes, with 60 members. The second extensive proteolytic group is a group of metalloproteases with 48 members. There are also 26 cysteine, 4 aspartic, 2 asparagine, 3 threonine, 2 unclassified peptidase members and 3 inhibitors. The subcellular localization of all annotated proteases was identified. In the membrane and inner fractions there were no differences in protease gene quantities between both strains (48 genes of membrane and 90 genes of intracellular proteases). For both *B. pumilus* genomes (7P and 3-19) 10 genes of secreted proteases were found. Among them, genes of serine proteases (subtilisin-like protease (*aprBp*) and glutamyl endopeptidase (*gseBp*) and metzincin metalloproteases (*mprBp*) were confirmed. The surroundings of extracellular protease genes have no differences for genomes of 7P and 3-19 strains.

**Figure 6:**
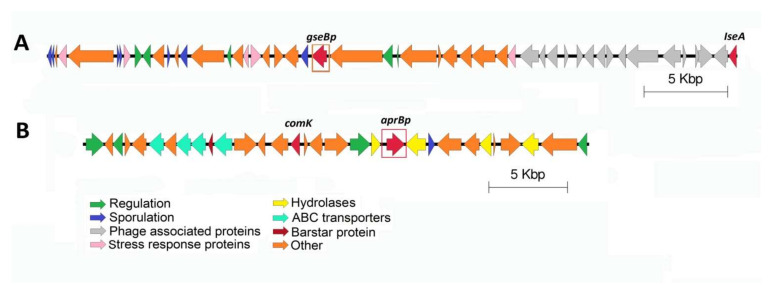
Alignment of genome loci containing gene of GseBp (**A**) and AprBp (**B**) proteases from *B. pumilus* 7P and 3-19 strains.

*B. pumilus* 7P and 3-19 secrete extracellular proteases during the stationary growth phase as part of their adaptation process, which allows cells to optimally use available resources and thereby ensure survival [56,57,58]. It is known that two-component systems and global regulatory factors play a role in the regulation of protease genes, which are closely related to biofilm formation [59] and the transition of cells to the stage of sporulation [60]. Using genetic analysis, the neighboring regions of the *B. pumilus* 7P and 3-19 extracellular protease genes were visualized. It has been shown that in the neighboring regions of the glutamyl endopeptidase gene, unlike other proteases, there are many spore proteins (Figure 6A).

At a distance of ~25 Kbp from the protease *gseBp* gene, the gene of IseA protease inhibitor was identified, which is part of the previously identified prophage Bp1. IseA protein is an inhibitor of autonomic DL-endopeptidases. Overexpression of IseA induces long-chain cell morphology and is induced by antibiotics targeting the cell wall [61,62]. The RapH protein gene was found near the Bp1 prophage region. RapH protein control sporulation and competence by acting on two distinct response regulator proteins: Spo0F and ComA, respectively [63]. It represses sporulation by dephosphorylating the intermediate response regulator Spo0F and inhibits competence by preventing ComA from binding to its target promoters [63].

The gene for the global regulator AbrB, which inhibits the expression of protease genes in the phase of exponential growth [64], was found surrounded by the subtilisin-like protease gene (Figure 6B). Next to the protease genes, the ComK competence protein, barstar protein, genes for ABC transporters and signal peptidase associated with the biogenesis of secreted proteins were discovered.

Based on the analysis of genome-wide sequencing data, it can be concluded that the degradomes of both strains 7P and 3-19 do not differ in content including 148 proteases of various catalytic classes, among which serine proteases are dominant. The subcellular localization of the annotated proteins of both strains corresponds to 90 intracellular, 48 membrane-bound, and 10 extracellular proteases. The genomic environment of each of the secreted proteases (subtilisin-like protease, glutamyl endopeptidase, and metalloprotease), isolated from the culture fluid of *B. pumilus* 7P and *B. pumilus* 3-19, remains unchanged.

### 3.7. Analysis of Single-Nucleotide Polymorphisms

**Figure 7:**
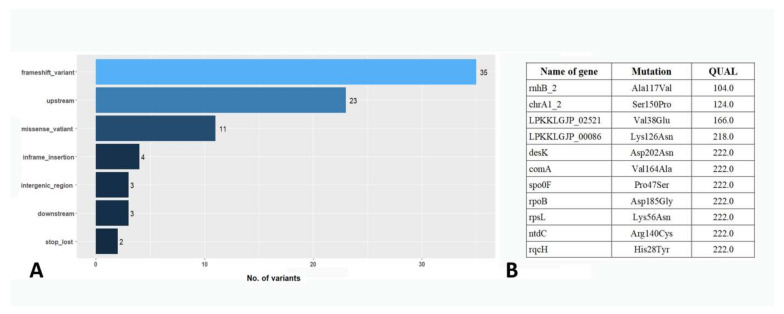
The ratio of nucleotide variants with different effects identified in the 3-19 strain genome (**A**). List of missense variants found in the genome (**B**).

To search for possible reasons for the increased secretion of hydrolytic enzymes in the *B. pumilus* 3-19 strain, a comparative analysis of variants in the genomes of 7P and 3-19 strains was performed. A total of 81 nucleotide variants were found in the 3-19 genome, which differed in the genome of the 7P strain (Figure 7A). Among them, 11 missense variants resulted in non-synonymous amino acid substitution in proteins (Figure 7B).

The presence of the mutation in codon 56 of the *rpsL* gene (Lys56Asn), which encodes the S12 ribosomal protein, was confirmed. The mutation occurred at the streptomycin binding site, as previously established, and caused resistance to high concentrations (>100 μg/mL) of streptomycin in the 3-19 strain [39]. The presence of the mutation in the *rpoB* gene (Asp185Gly), encoding the RNA polymerase β subunit, was also shown. Mutations in this gene are known to confer bacterial resistance to the antibiotic rifampicin [65]. However, the disk diffusion method showed the absence of this resistance in 3-19 strain [39]. The remaining variants were analyzed in terms of their influence on changes in hydrolase activity in the *B. pumilus* 3-19 strain. Among the identified variants, no changes were found in the proteases, phosphatase and ribonuclease genes, as well as in the upstream regions of these genes (Table S6). Analysis of high-quality variants (QUAL > 200.0) revealed missense mutations in the *comA*, *spo0F* genes, and in the upstream region of the *srlR* gene. These genes play an important role in the development of competence, sporulation, and the biosynthesis of extracellular enzymes during the stationary phase of culture growth. A mutation in the comA gene was identified in the DNA-binding helix-turn-helix (HTH) domain. This C-terminal domain shares sequence homology with LuxR and is involved in the binding of the ComA protein to promoters of target genes [66]. A mutation in the DNA-binding domain could affect the efficiency of binding of the ComA protein to promoters of target genes, such as the degQ gene, which in turn is involved in the regulation of hydrolase synthesis through DegU [67]. In addition, a mutation was found in the upstream region of the slrR gene, which could also affect the expression of this gene product. The SlrR protein interacts directly with the transcription factor DegU, which affects the expression of hydrolase genes [67]. In general, the point substitutions in *comA*, *spo0F*, *slrR* genes that we have identified can affect the formation of signal transduction networks for the genes of *B. pumilus* 3-19 extracellular hydrolases, the phenotypic effect of which we observe.

## 4. Conclusions

Whole-genome sequencing of the *B. pumilus* 7P soil isolate and its streptomycin-resistant mutant *B. pumilus* 3-19 allowed us to detect plasmid DNA (pDA7) in the 7P strain of 6019 bp, which is absent in the 3-19 strain. The functional annotation of the genomes of strains 7P and 3-19 showed approximately the same number of CDS for both strains (3794 and 3746, respectively). Proteins of cell defense mechanisms are widely represented, as well as toxins with nuclease activity to limit the growth of competitors. Pan-genomic analysis of five phylogenetically close strains of *B. pumilus*, including strains 7P and 3-19, showed a core of 3268 CDS and revealed 72 unique proteins for strains 7P and 3-19. Among the unique proteins, prophage proteins associated with transposition and horizontal gene transfer have been identified. The presence of unique CDS due to the inclusion of prophage DNA regions in the 7P and 3-19 genomes indicates differences with the structure of the most phylogenetically close genomes and may be associated with increased resistance of these strains to the transformation of exogenous DNA. Both strains, 7P and 3-19 do not differ in the composition of the degradome, which includes 148 protease genes of different classes. The environment of the genes of extracellular proteases, the secretion of which differs in the two strains, showed the absence of any structural rearrangements. Analysis of nucleotide polymorphisms showed the presence of 81 variants in the genome of strain 3-19. The presence of a mutation in the *rpsL* gene that led to streptomycin resistance was shown. The point substitutions in *comA*, *spo0F*, *slrR* genes have been identified and may be associated with the appearance of high expression of hydrolytic enzyme genes in strain 3-19. The genomic information presented in this study reveals the structural features of the genomes of *B. pumilus* 7P and 3-19 strains and can help us in the future to more effectively use these strains for the production of biotechnologically important enzymes.

## Figures and Tables

**Figure 1:**
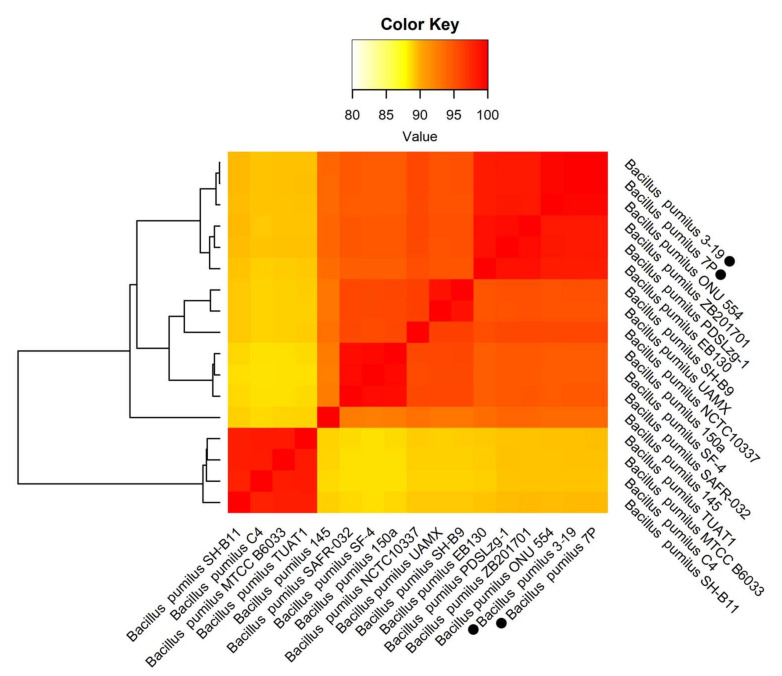
Heatmap illustrates the results of ANI analysis between representatives of *B. pumilus*. Variation percent of identity is shown on the color scale.

**Table 1:** Characteristics of *B. pumilus* 7P and 3-19 strains. Genome assembly and annotation statistics of both bacteria.

Strains	Year Isolated	Location Isolated	Description	GenBank Accession Number	Total Length (bp)	No. of ORFs	Coverage
*B. pumilus* 7P	1974	Russia	Soil isolate	CP058911.1	3,609,117	3662	42.0×
*B. pumilus* 3-19	1994	Russia	Derivative of 7P	CP054310.1	3,609,444	3679	50.0×

## Data Availability

The data used to support the findings of this study are included within the article.

## Supplementary Materials

The following supporting information can be downloaded at: https://www.mdpi.com/article/10.3390/genes13030409/s1, Figure S1: Electrophoresis of plasmid DNA from *B. pumilus* 7P. (1) M12 marker (10 Kb); (2) Negative control; (3) DNA of putative plasmid. Table S1: Enzymatic activity of B. pumilus 7P and 3-19 strains. The 7P and streptomycin-resistant isolate 3-19 were tested on the ability to hydrolyze different substrates: para-nitrophenyl phosphate (pNPP), 2% casein, Z-Glu-pNA. Table S2: Accession numbers of available *B. pumilus* complete genomes. Table S3: Average Nucleotide Identity (ANI) values between available whole-genome sequences from *B. pumilus* (accessed September 2021). Table S4: List of *B. pumilus* 7P/3-19 strains specific proteins versus strains ONU 554, PDSLzg-1, ZB201701. Table S5: List of *B. pumilus* 7P/3-19 strains unique CDSs. Table S6: List of variants in the *B. pumilus* 3-19 strains genome.
