# Supplementary material for: Comparative Genome Analysis of Two Bacillus pumilus Strains Producing High Level of Extracellular Hydrolases
Source: Genes (Basel). 2022 Feb 24;13(3):409. doi: 10.3390/genes13030409 (PMC8950961; doi:10.3390/genes13030409)

Figure S1. Electrophoresis of plasmid DNA from *B. pumilus* 7P. (1) M12 marker (10 Kb); (2) Negative control; (3) DNA of putative plasmid.

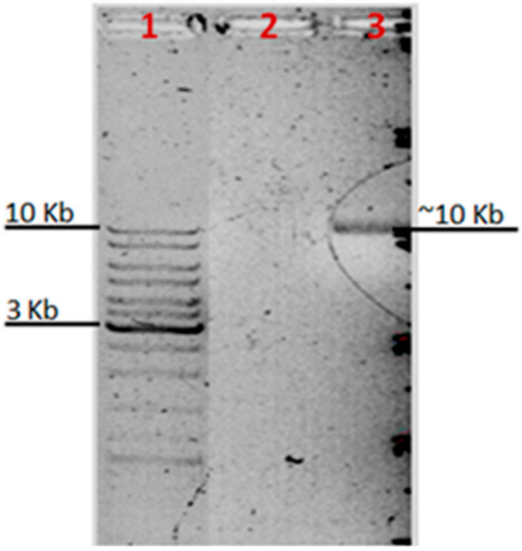

Supplement: Supplementary file 1 [file genes-13-00409-s001.zip › Figure S1.pdf]
