# Supplementary material for: Comparative Genome Analysis of Two Bacillus pumilus Strains Producing High Level of Extracellular Hydrolases
Source: Genes (Basel). 2022 Feb 24;13(3):409. doi: 10.3390/genes13030409 (PMC8950961; doi:10.3390/genes13030409)
Supplement: Supplementary file 1 [file genes-13-00409-s001.zip › Table S1.pdf]

Table S1. Enzymatic activity of B. pumilus 7P and 3-19 strains. The 7P and streptomycin-resistant isolate 3-19 were tested on the ability to hydrolyze different substrates: para-nitrophenyl phosphate (pNPP), 2% casein, Z-Glu-pNA.

***B. pumilus* strains**	**Enyzme activity (U/ml)**			
	**RNA**	**pNPP**	**Caseinolytic activity**	**Z-Glu-pNA**
7P (wild type)	2,500	2	42	4.8
3-19	20,000	7,010 ± 0,09	90	16
