# Supplementary material for: Comparative Genome Analysis of Two Bacillus pumilus Strains Producing High Level of Extracellular Hydrolases
Source: Genes (Basel). 2022 Feb 24;13(3):409. doi: 10.3390/genes13030409 (PMC8950961; doi:10.3390/genes13030409)
Supplement: Supplementary file 1 [file genes-13-00409-s001.zip › Table S2.pdf]

Table S2. Accession numbers of available *B. pumilus* complete genomes.

№	Bacterial strains	GenBank accession number
1	*Bacillus pumilus* SF-4	NZ_CP047089.1
2	*Bacillus pumilus* C4	NZ_CP011109.1
3	*Bacillus pumilus* 3-19	CP054310.1
4	*Bacillus pumilus* 7P	CP058911.1
5	*Bacillus pumilus* NCTC10337	NZ_LT906438.1
6	*Bacillus pumilus* SH-B11	NZ_CP010997.1
7	*Bacillus pumilus* UAMX	NZ_CP058951.1
8	*Bacillus pumilus* SH-B9	NZ_CP011007.1
9	*Bacillus pumilus* MTCC B6033	NZ_CP007436.1
10	*Bacillus pumilus* 150a	NZ_CP027034.1
11	*Bacillus pumilus* TUAT1	NZ_AP014928.1
12	*Bacillus pumilus* PDSLzg-1	NZ_CP016784.1
13	*Bacillus pumilus* ONU 554	NZ_CP060799.1
14	*Bacillus pumilus* ZB201701	NZ_CP029464.1
15	*Bacillus pumilus* SAFR-032	NC_009848.4
16	*Bacillus pumilus* 145	NZ_CP027116.1
17	*Bacillus pumilus* EB130	CP081199.1
