# Supplementary material for: Comparative Genome Analysis of Two Bacillus pumilus Strains Producing High Level of Extracellular Hydrolases
Source: Genes (Basel). 2022 Feb 24;13(3):409. doi: 10.3390/genes13030409 (PMC8950961; doi:10.3390/genes13030409)
Supplement: Supplementary file 1 [file genes-13-00409-s001.zip › Table S4.pdf]

Table S4. List of *B. pumilus* 7P/3-19 strains specific proteins versus strains ONU 554, PDSLzg-1, ZB201701

peptidoglycan DD-metalloendopeptidase family protein
BMP family protein
N-acetylmuramoyl-L-alanine amidase
PAS domain S-box protein
amino acid ABC transporter ATP-binding protein
PBP1A family penicillin-binding protein
IS5/IS1182 family transposase
phosphoribosyltransferase domain-containing protein
collagen-like triple helix repeat-containing protein
MerR family DNA-binding transcriptional regulator
DNA topoisomerase IV subunit B
DUF1453 family protein
cytochrome P450
DUF4917 family protein
DNA primase
phage antirepressor KilAC domain-containing protein
ORF6N domain-containing protein
phosphotransferase
tetratricopeptide repeat protein
ATP-binding cassette domain-containing protein
MOSC domain-containing protein
orthopoxovirus pf05708 family protein
spore protease YyaC
DUF3298 domain-containing protein
transcription termination factor Rho
cellulose biosynthesis cyclic di-GMP-binding regulatory protein BcsB
hypothetical protein
DUF4258 domain-containing protein
transposase
hypothetical protein
NPP1 family protein
hypothetical protein
DUF3800 domain-containing protein
MaoC family dehydratase
RapH phosphatase inhibitor
hypothetical protein
DUF3732 domain-containing protein
DUF4241 domain-containing protein
hypothetical protein
myo-inosose-2 dehydratase
sugar porter family MFS transporter
sugar phosphate isomerase/epimerase
3D-(3,5/4)-trihydroxycyclohexane-1,2-dione acylhydrolase (decyclizing)

5-deoxy-glucuronate isomerase
5-dehydro-2-deoxygluconokinase
CoA-acylating methylmalonate-semialdehyde dehydrogenase
MFS transporter
Gfo/Idh/MocA family oxidoreductase
sugar phosphate isomerase/epimerase
hypothetical protein
hypothetical protein
transglycosylase
DUF4935 domain-containing protein
hypothetical protein
hypothetical protein
hypothetical protein
Fur-regulated basic protein FbpA
ERCC4 domain-containing protein
hypothetical protein
DUF669 domain-containing protein
AAA family ATPase
host-nuclease inhibitor Gam family protein
hypothetical protein
DUF2513 domain-containing protein
helix-turn-helix domain-containing protein
helix-turn-helix transcriptional regulator
tyrosine-type recombinase/integrase
hypothetical protein
class II fructose-1,6-bisphosphate aldolase
ImmA/IrrE family metallo-endopeptidase
polysaccharide deacetylase
amidinotransferase
